# Characterization of population-based variation and putative functional elements for the multiple-cancer susceptibility loci at 5p15.33

**DOI:** 10.12688/f1000research.5186.1

**Published:** 2014-10-02

**Authors:** Lisa Mirabello, Charles C. Chung, Meredith Yeager, Sharon A Savage

**Affiliations:** 1 Division of Cancer Epidemiology and Genetics, National Cancer Institute,National Institutes of Health, Department of Health and Human Services, Bethesda, MD 20892, USA; 2 Cancer Genomics Research Laboratory, National Cancer Institute, Division of Cancer Epidemiology and Genetics, Leidos Biomedical Research, Inc., Frederick, MD 20877, USA

**Keywords:** TERT, CLPTM1L, population genetics, 5p15.33

## Abstract

**Background:**

*TERT* encodes the telomerase reverse transcriptase, which is responsible for maintaining telomere ends by addition of (TTAGGG) _n_ nucleotide repeats at the telomere.  Recent genome-wide association studies have found common genetic variants at the *TERT-CLPTM1L* locus (5p15.33) associated with an increased risk of several cancers.

**Results:**

Data were acquired for 1627 variants in 1092 unrelated individuals from 14 populations within the 1000 Genomes Project.  We assessed the population genetics of the 5p15.33 region, including recombination hotspots, diversity, heterozygosity, differentiation among populations, and potential functional impacts. There were significantly lower polymorphism rates, divergence, and heterozygosity for the coding variants, particularly for non-synonymous sites, compared with non-coding and silent changes. Many of the cancer-associated SNPs had differing genotype frequencies among ancestral groups and were associated with potential regulatory changes.

**Conclusions:**

Surrogate SNPs in linkage disequilibrium with the majority of cancer-associated SNPs were functional variants with a likely role in regulation of *TERT* and/or *CLPTM1L. * Our findings highlight several SNPs that future studies should prioritize for evaluation of functional consequences.

## Introduction

The 5p15.33 locus includes the *TERT* (human telomerase reverse transcriptase) and the *CLPTM1L* (alias CRR9; cleft lip and palate transmembrane 1 like) genes. Telomerase reverse transcriptase (TERT) is the essential catalytic component of the telomerase holoenzyme responsible for maintaining telomere ends. Telomerase compensates for DNA polymerase’s inability to fully replicate the lagging DNA strand by adding hexanucleotide (5'-TTAGGG-3') _n_ repeats to the 3’ end of chromosomes using a template sequence within the RNA component (TERC) of the enzyme ^1^. Telomeres, consisting of these hexanucleotide repeats and several associated proteins, are responsible for preserving chromosomal stability by protecting chromosomes from end-to-end fusion, atypical recombination, and degradation ^2^. In normal differentiated cells, expression of telomerase is very low or absent and telomeres erode by 50 to 200 base pairs with each cell division ^1^. When the telomeres become critically short, they act as a cellular clock and signal cellular senescence and apoptosis ^3, 4^. In contrast, telomerase activity has been detected in 90% of human cancers ^5, 6^ and allows these malignant cells to continually divide by bypassing cellular crisis ^7^.

*CLPTM1L* is located approximately 23 kilobases (kb) centromeric of *TERT*. Little is known about the function of the *CLPTM1L* protein. It is a predicted transmembrane protein that is expressed in a range of normal and malignant tissues including skin, lung, breast, ovary and cervix, and has been shown to sensitize ovarian cancer cells to cisplatin-induced apoptosis ^8^.

The clinically related telomere biology disorders (TBDs), such as pulmonary fibrosis or aplastic anemia, are associated with germline mutations causing amino acid substitutions, additions, deletions, and frame shift mutations within *TERT* ^9, 10^. Patients with the more severe TBD, dyskeratosis congenita (DC) have very high risks of bone marrow failure and cancer, and have telomeres below the 1 ^st^ percentile for their age ^11^. DC represents the most clinically severe outcome of germline *TERT* mutations and often presents in childhood. Individuals with isolated aplastic anemia or pulmonary fibrosis due to *TERT* mutations tend to manifest clinical symptoms in adulthood.

Genome-wide association studies (GWAS) have found that common genetic variants, in the form of single nucleotide polymorphisms (SNPs), within the *TERT-CLPTM1L* locus (5p15.33) are associated with relatively low but highly statistically significant risks (odds ratios for risk alleles ranging between 1.05–1.6) of several cancers, including glioma ^12, 13^, basal cell carcinoma ^14, 15^, testicular ^16^, pancreatic ^17^, lung ^18– 20^, bladder ^21^, colorectal ^22^, breast ^23^, and overall cancers ^24^ [reviewed in ^25, 26^].

Both *TERT* and *CLPTM1L* are evolutionarily conserved across diverse species, which suggests their functional importance ^8, 27, 28^. *TERT* has low nucleotide diversity, and common SNPs in this gene region show low levels of differentiation among populations and high ancestral allele frequencies ^28, 29^; this pattern of low overall diversity suggests that *TERT* may be constrained ^29^.

The 1000 Genomes Project Consortium has reported that different populations have different profiles of rare and common variants; and, varying degrees of purifying selection at functionally relevant low-frequency sites which lead to substantial local population differentiation ^30^. Large surveys of human genetic variation have described an excess of rare genetic variants as a result of a recent population expansion and weak purifying selection ^31– 33^, particularly for variants in disease genes and for individuals of European ancestry ^33^.

In order to better understand the population genetics underlying the 5p13.3 locus associated with cancer, we conducted a detailed analysis of allele frequency patterns among ancestral group, levels of differentiation, and recombination at the 5p15.33 locus using 1000 Genomes Project ^34^ data. We retrieved data for the *TERT-CLPTM1L* genes and flanking regions for 1092 individuals from 14 populations. Analyses were focused on understanding how allele frequencies differ between populations, and evaluation of the cancer-associated SNPs and their surrogate markers for potential functional elements.

## Materials and methods

### Dataset

Data were retrieved for 1627 variants on 5p15.33 (hg19, chr5: 1,243,287–1,355,002) for all individuals in the 14 populations (1092 individuals) included in the 1000 Genomes project (2012 February release) ^34^. Eighteen potentially related individuals were removed, which resulted in 1074 individuals. We also retrieved data for a flanking region, approximately 10kb upstream and downstream, in order to improve understanding of these gene regions [ Data File 1].

### Data analysis

The package ARLEQUIN version 3.5 ^35^ was used to compute *F*_ST_ values, diversity, AMOVA, and heterozygosity. *F*_ST_ values based on allele frequencies were calculated as a measure of population differentiation, and significance was estimated with 10,000 permutations; and, these levels were compared to the genome-wide average for autosomal SNPs ( *F*_ST_ ≈ 0.1 ^36– 39^). The population of African-Americans in the Southwestern United States (ASW) was grouped with the two populations of West African ancestry (Luhya in Kenya [LWK] and Yoruba in Nigeria [YRI]) since in our population level analyses they were found to be most closely related to these individuals of African ancestry, as previously observed ^40^. In order to apportion the fraction of the genetic variance due to differences between and within ancestral groups (European, East Asian, West African, and American) and infer the genetic structure of the populations, AMOVA was performed with 10,000 permutations. HAPLOVIEW version 4.1 ^41^ was used to determine the degree of linkage disequilibrium (LD) and minor allele frequency (MAF). The GLU genetics’ ld.tagzilla module was used for the tag analysis with a LD pairwise r ^2^ threshold of 0.8. Pairwise LD was analyzed separately for the four ancestral groups and used to select tag SNPs for each region.

SNPs within *TERT* and *CLPTM1L* were grouped by functional category ( *i.e.*, coding *vs*. non-coding, and synonymous *vs*. non-synonymous variants), and tested for significant differences in the normalized number of variant sites, allelic frequency divergence, heterozygosity, minor allele frequency (MAF), and levels of differentiation among populations; significant differences would suggest that these functional categories of loci were not affected similarly, as expected under the assumption of neutrality. The allelic frequency divergence between ancestral groups was computed using: *d* = 1-[( *x*_1_ *y*_1_) ^1/2^ + ( *x*_2_ *y*_2_) ^1/2^], where *x*_1_ and *y*_1_ are the frequencies of the first allele and *x*_2_ and *y*_2_ are the frequencies of the second allele ^42^. The normalized number of variant sites was calculated as: θ^ = K/Σ ^n-1^_i=1_ i ^-1^L, where K is the number of variant sites, n is the number of chromosomes, and L is the total sequence length. Differences between the SNP functional categories were tested for significance with a two-tailed *t*-test. SIFT ( **S**orts **I**ntolerant **F**rom **T**olerant) and Polyphen 2 ( **Poly**morphism **Phen**otyping v **2**) were used to predict the potential impact of an amino acid substitution ^43, 44^.

To identify recombination hotspots in this region, we used SequenceLDhot ^45^, a program that uses the approximate marginal likelihood method ^46^ and calculates likelihood ratio statistics at a set of possible hotspots. We used the four ancestral groups [European (EUR; n=379), East Asian (EA; n=286), American (AM; n=184), and African (AFR; n=246)] to calculate background recombination rates using PHASE v2.1 ^47, 48^. The likelihood ratio statistics of 12 predicts the presence of a hotspot with a false-positive rate of 1 in 3,700 independent tests.

Putative functional elements were assessed using the UCSC genome browser ( http://genome.ucsc.edu/), a publically available bioinformatics website, for ENCODE Regulation and Comparative Genomics tracks for all of the cancer-associated SNPs and their surrogates for each ancestral group. SNPs were considered surrogates for cancer-associated SNPs for each ancestral group if the r ^2^ ≥0.60, the inter-marker distance ≤200kb, and the MAF ≥0.05. We assessed potential regions of open chromatin with DNase hypersensitivity; potential regulatory histone marks (H3K4Me1, H3K4Me3, H3K27Ac); protein binding sites; regulatory motifs; CpG islands; conserved mammalian microRNA regulatory binding sites; and evolutionary conservation among placental mammals using the phylop basewise conservation measurement ^49^. Functional elements were also assessed using RegulomeDB, an integrated database that annotates SNPs with known or predicted regulatory DNA elements, including DNase hypersensitivity, transcription factor binging sites, and promoter regions that regulate transcription using data from GEO, ENCODE, and published literature ^50^. RegulomeDB scores are a heuristic scoring system based on confidence that a variant is located in a functional region and likely results in a functional consequence, these are used to assist comparison among annotations ^50^. Lower scores indicate increased evidence; category 2 scores are variants likely to affect binding, category 3 scores are less likely to affect binding; and 4, 5, or 6 scores are variants with minimal binding evidence.

## Results

Genotype data for 1627 variants on 5p15.33 (hg19, chr5: 1,243,287–1,355,002) for 1074 individuals from 14 populations Data were retrieved for 1627 variants on 5p15.33 (hg19, chr5: 1,243,287–1,355,002) for all individuals in the 14 populations (1092 individuals) included in the 1000 Genomes project (2012 February release). Eighteen potentially related individuals were removed, which resulted in 1074 individuals. Click here for additional data file. Copyright: © 2014 Mirabello L et al. 2014 Data associated with the article are available under the terms of the Creative Commons Zero "No rights reserved" data waiver (CC0 1.0 Public domain dedication).

### Allele frequency spectrum

There were 1627 variants in the *TERT-CLPTM1L* region among all individuals (N=1074): 167 were upstream of *TERT*, 563 in *TERT* (including UTR, intronic and exonic regions), 353 were between *TERT* and *CLPTM1L* (downstream of *TERT* and upstream of *CLPTM1L*), 412 in *CLPTM1L* (including UTR, intronic and exonic regions), and 132 downstream of *CLPTM1L*. A summary of the variation for the different functional categories of polymorphisms in *TERT* and *CLPTM1L* is given in Table 1. The majority of SNPs in *TERT* and *CLPTM1L* were in intronic regions (N=903), only 72 were exonic (49 in *TERT* and 18 in *CLPTM1L*). 46 of the exonic variants were synonymous changes (32 in *TERT* and 9 in *CLPTM1L*) and 26 were non-synonymous protein altering variants (PAV) (17 in *TERT* and 9 in *CLPTM1L*). The SNPs previously associated with cancer at 5p15.33 ^25^ are all located in the intronic regions of *TERT* or *CLPTM1L* or intergenic between these genes, except for one which is a coding synonymous SNP in *TERT* (rs2736098; Table 2).

**Table 1.** Summary of variation for the different classes of polymorphisms for all individuals (n=1074).

Polymorphism type	bp screened	No. Polys	Frequency (SNP/bp)	θ^	Het.	MAF
Non-coding*	61,757	903	1/68	1.77E ^-03^	0.120	9.03%
Coding	7,126	72	1/99	1.22E ^-03^	0.036	2.14%
Synonymous		46	1/155	7.82E ^-04^	0.048	2.92%
Non-synonymous		26	1/274	4.42E ^-04^	0.014	0.69%

* includes intronic and 3' UTR SNPs; bp = base-pairs; Polys = polymorphisms; θ^ = normalized number of variant sites; Het. = heterozygosity; MAF = minor allele frequency; *F*_ST_ = level of differentiation among ancestral groups.

**Table 2.** Summary of the cancer-associated SNPs at the *TERT-CLPTM1L* locus.

SNP	Position	Gene	Function	Ethnicity ^†^	Cancer(s)	Alleles ^‡^	RAF				*F*_ST_
							AFR	EUR	AM	EA	
rs4246742	1267356	*TERT*	intron	Misc.	Lung	T:A	67.4%	83.5%	77.7%	60.7%	0.055
rs10069690	1279790	*TERT*	intron	EUR, AFR	Breast	C: T	62.7%	27.5%	25.1%	15.9%	0.17
rs2242652	1280028	*TERT*	intron	EUR	Prostate	G: A	14.4%	21.0%	18.1%	16.4%	0.003
rs13167280	1280477	*TERT*	intron	EUR	Bladder	G: A	2.8%	13.0%	13.8%	19.1%	0.036
rs2736100	1286516	*TERT*	intron	Misc, EUR, Asian	Lung, CNS, Bladder, Pancreas, Testis	A: C	43.8%	50.0%	44.6%	39.3%	0.009
rs2853676	1288547	*TERT*	intron	Misc.	CNS, Lung	C: T	21.2%	27.5%	26.8%	16.1%	0.016
rs2736098	1294086	*TERT*	coding, syn.	Misc.	Bladder, Lung	C: T	6.0%	23.4%	19.5%	32.9%	0.062
rs2736108	1297488	Intergenic		EUR	Breast	C: T	6.7%	27.5%	22.3%	25.9%	0.045
rs2853668	1300025	Intergenic		EUR, Misc.	Pancreas, Lung, Colon	G: T	52.6%	25.8%	30.8%	24.3%	0.069
rs2735845	1300584	Intergenic		Misc.	Lung	C: G	4.9%	20.1%	24.9%	30.1%	0.055
rs4635969	1308552	Intergenic		Misc., EUR	Lung, Pancreas, Testis	G: A	34.1%	19.3%	12.7%	12.1%	0.055
rs4975615	1315343	Intergenic		Misc.	Lung	A: G	49.4%	42.3%	28.3%	16.3%	0.088
rs4975616	1315660	Intergenic		Misc., EUR	Lung, Pancreas, Testis	A: G	72.1%	44.3%	31.9%	16.3%	0.201
rs1801075	1317949	Intergenic	near gene 3'	Misc.	Lung	T: C	14.0%	19.1%	15.8%	4.4%	0.035
rs451360	1319680	*CLPTM1L*	intron	Misc., EUR	Lung	C: A	2.6%	21.6%	14.1%	11.9%	0.053
rs380286	1320247	*CLPTM1L*	intron	Misc.	Lung	G: A	61.6%	45.4%	35.6%	13.6%	0.156
rs402710	1320722	*CLPTM1L*	intron	Misc., EUR, Asian	Bladder, Lung	C: T	46.8%	35.5%	32.8%	29.4%	0.017
rs401681	1322087	*CLPTM1L*	intron	Misc, EUR, Asian	Bladder, Prostate, Pancreas, BCC, Melanoma, SCC, Lung	C: T	58.6%	45.9%	42.7%	30.4%	0.048
rs465498	1325803	*CLPTM1L*	intron	Misc, Asian	Lung	A: G	57.9%	46.2%	35.0%	16.4%	0.124
rs452932	1330253	*CLPTM1L*	intron	Misc.	Lung	T: C	58.2%	46.2%	35.6%	15.7%	0.128
rs452384	1330840	*CLPTM1L*	intron	Misc.	Lung	T: C	58.2%	45.9%	35.6%	15.7%	0.128
rs467095	1336221	*CLPTM1L*	intron	Misc.	Lung	T: C	71.2%	46.3%	35.9%	15.9%	0.194
rs31489	1342714	*CLPTM1L*	intron	Misc., EUR, Asian	Lung, Pancreas, Testis	C: A	47.2%	43.1%	31.4%	15.7%	0.084

^†^ Ethnicity as reported in Mocellin *et al.* (2012); ^‡^ major allele:minor allele, and the risk allele is underlined; syn. = synonymous change; RAF = risk allele frequency; *F*_ST_ = level of differentiation among ancestral groups; misc. = miscellany, indicating a mix of different races; AFR = African ancestry; EUR = European ancestry; AM = American ancestry; EA = East Asian ancestry.

**Figure 1.**
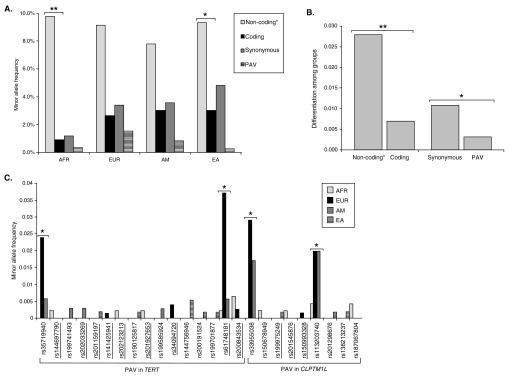
Variation in *TERT-CLPTM1L* by ancestral group. ( **A.**) Average minor allele frequency of the polymorphisms by functional category for each group; ( **B.**) average level of differentiation among ancestral groups ( *F*_ST_) for the polymorphisms by functional category; ( **C.**) minor allele frequency of each protein-altering variant by ancestral group, the underlined variants are predicted to be potentially deleterious with SIFT and/or Poly-Phen. ** indicates a significant difference with a *P* <0.01, * *P* <0.05. PAV = non-synonymous protein-altering variation; AFR = African ancestry; EUR = European ancestry; AM = American ancestry; EA = East Asian ancestry.

Since there were so few coding variants in the *TERT* and *CLPTM1L* loci, we combined them for the following analyses. The normalized number of variant sites, heterozygosity, and MAFs were significantly different by functional SNP category in *TERT* and *CLPTM1L* ( *P* values <0.01; Table 1). Specifically, the non-coding SNPs (compared with coding SNPs) and synonymous SNPs (compared with non-synonymous SNPs) had significantly higher numbers of variant sites, heterozygosity, and MAFs ( Table 1). These trends were consistent in all ancestral groups ( Figure 1A). The most significant differences between coding and non-coding SNPs were in African populations (non-coding average MAF 9.8% *vs.* coding average MAF 0.9%); and, the most significant differences between synonymous (syn.) *versus* non-synonymous (non-syn.) SNPs were in East Asian populations (syn. average MAF 4.8% *vs.* non-syn. average MAF 0.2%) ( Figure 1A). There were significantly different levels of differentiation among ancestral groups for coding *versus* non-coding and synonymous *versus* non-synonymous SNPs ( Figure 1B).

### Protein altering variation

All PAVs were present at a rare or low frequency ( Figure 1C). European ancestry individuals had higher MAFs for many of the PAVs in *TERT* and *CLPTM1L*, and there were significant MAF differences among ancestral groups for rs35719940, rs61748181, rs33955038, and rs113203740 ( Figure 1C). Nine (53%) of the 17 PAVs observed in *TERT* and three (33%) of the nine PAVs observed in *CLPTM1L* were reported to be damaging by Polyphen and/or SIFT (two *in silico* approaches; underlined in Figure 1C). Most of these potentially damaging variants were only observed in one individual. However, three possibly damaging variants in *TERT* were observed in multiple individuals [rs34094720 (N=3), rs61748181 (N=31), rs200843534 (N=5)] ( Figure 1C).

### Patterns of diversity and recombination among ancestral groups

**Figure 2.**
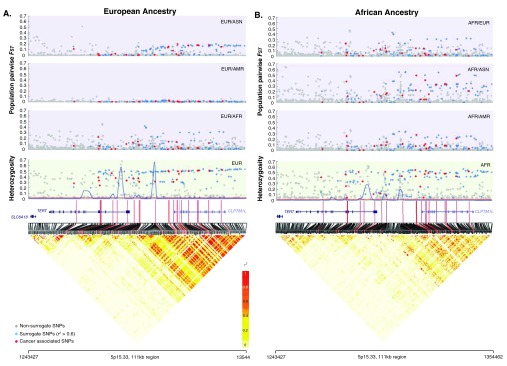
Summary of population genetics parameters in European ( **A.**) and African ( **B.**) ancestry individuals for 5p15.33. Linkage disequilibrium (LD), recombination hotspots, heterozygosity, and pairwise *F*_st_ values are shown for the cancer-associated SNPs (red dots), surrogate SNPs (blue dots), and non-surrogate SNPs (grey dots). LD pattern (see color legend) is shown for SNPs with a MAF ≥0.05. The red lines represent an extension of the location of the cancer-associated SNPs. The blue lines in the heterozygosity plot indicate the location of the recombination hotspots. For the pairwise *F*_st_ estimates, the populations are indicated in the top corner of each graph. AFR = African ancestry; EUR = European ancestry; AM = American ancestry; ASN = East Asian ancestry.

A summary of the variation by ancestral group for this region is given in Table 3. There was low nucleotide diversity (average of 5.0E ^-4^) by ancestral group and low differentiation among ancestral groups (90.4% of loci in this region had low *F*_ST_ <0.10; median *F*_ST_ = 0.005) (data not shown). The median *F*_ST_ among ancestral groups (AG) and within populations (WP) for SNPs located within *TERT* and *CLPTM1L* were low (AG *F*_ST_ = 0.0039 and 0.0040, respectively; and, WP *F*_ST_ = 0.0078 and 0.0091, respectively). The greatest level of pairwise differentiation was among African and East Asian ancestry populations (pairwise *F*_ST_ = 0.208), and among European and East Asian ancestry populations (pairwise *F*_ST_ = 0.104) ( Figure 2 and Supplementary Figure 1). The lowest level of pairwise differentiation was among European and American ancestry populations (pairwise *F*_ST_ = 0.01). The MAFs and heterozygosity estimates for SNPs in this region in European and American ancestry populations were highly correlated (r ^2^ = 0.95 and 0.965, respectively).

**Table 3.** Summary of the diversity at 5p15.33 by ancestral group.

	African (AFR)	European (EUR)	American (AM)	East Asian (EA)
No. individuals	233	378	177	286
No. polymorphic loci	1009	732	808	503
Heterozygosity (SD)	0.120 (0.16)	0.127 (0.18)	0.111 (0.16)	0.129 (0.16)
Nucleotide diversity	6.5E ^-04^	5.0E ^-04^	4.9E ^-04^	3.8E ^-04^

SD = standard deviation.

There was little to no LD in the *TERT* gene region but high LD was present in the *CLPTM1L* gene region ( Figure 2 and Supplementary Figure 1). There were 4–5 main recombination hotspots in *TERT* and between *TERT* and *CLPTM1L*, there were no hotspots located within *CLPTM1L* ( Supplementary Table 1) *.* The greatest recombination was observed in individuals with African ancestry (5 recombination hotspots), and the lowest recombination in individuals with East Asian ancestry (4 recombination hotspots and lower likelihood ratio statistics) ( Figure 2 and Supplementary Figure 1).

### Cancer-associated SNPs

Twenty-three SNPs significantly associated with cancer at 5p15.33 ^25^ were included in the analysis ( Table 2). Many of the cancer associated SNPs in this region had differing allele frequencies and heterozygosity among ancestral groups and populations, and had *F*_ST_ values close to or greater than 0.1 ( Table 2 and Supplementary Table 4). The risk allele was the rare allele at all of these SNPs, except at rs4246742 (associated with lung cancer; Table 2). Most of the cancer-associated SNPs in the *CLPTM1L* gene region are in regions of high LD, and therefore, have many surrogates (25–54 surrogate SNPs) with r ^2^ ≥0.6 ( Table 4 and Supplementary Table 2). In contrast, most of the SNPs in the *TERT* gene region are in a region of low LD and have no or few surrogates (0–5 surrogate SNPs) with r ^2^ ≥0.6 ( Table 4 and Supplementary Table 2). In East Asian ancestry individuals SNPs in the *CLPTM1L* gene region are particularly highly correlated, even some of the SNPs within *TERT* are in high LD in these individuals ( *i.e.*, rs10069690, rs2242652, and rs13167280; Supplementary Figure 1).

**Table 4.** Previously reported multiple-cancer susceptibility loci at 5q15.33 and their surrogates at an r ^2^ ≥0.6 and regulatory elements.

Locus			Surrogates ^†^				H3K4 Me1	H3K4 Me3	H3K27 Ac	DNase	Regulatory motifs altered	Proteins bound	CpG island	Regulome DB score	Mammal Conserv.
			AFR	EUR	AM	EA									
rs4246742	1267356	*TERT*	0	0	1	1				• (3)				5	
rs10069690	1279790	*TERT*	2	1	0	2				• (19)				5	
rs2242652	1280028	*TERT*	3	1	1	1				• (17)	HEN1, ZFX, E2A, REST			5	
rs13167280	1280477	*TERT*	0	0	1	0				• (19)	NKX2			5	
rs2736100	1286516	*TERT*	3	0	8	9				• (4)				5	
rs2853676	1288547	*TERT*	0	0	1	1								5	
rs2736098	1294086	*TERT*	3	2	2	4	• (4)	• (4)		• (8)	NRSF, LRF		•	5	
rs2736108	1297488	Intergenic	3	2	3	3	• (3)			• (25)		EBF1		4	
rs2853668	1300025	Intergenic	0	0	1	1				• (2)				5	
rs2735845	1300584	Intergenic	0	2	2	3								—	•
rs4635969	1308552	Intergenic	13	4	3	45	• (2)		• (2)		FOXO1, SOX15			6	
rs4975615	1315343	Intergenic	24	48	48	54	• (8)	• (4)	• (4)		ZBTB3			5	
rs4975616	1315660	Intergenic	9	47	38	54	• (11)	• (5)	• (4)	• (8)				5	
rs1801075	1317949	Intergenic	2	6	6	0								—	
rs451360	1319680	*CLPTM1L*	0	7	4	52				• (4)	HIC1, OLF-1			5	
rs380286	1320247	*CLPTM1L*	18	47	47	47			• (3)	• (3)				5	
rs402710	1320722	*CLPTM1L*	20	8	0	0			• (3)		HEN1			5	
rs401681	1322087	*CLPTM1L*	25	46	21	0			• (3)	• (6)				5	
rs465498	1325803	*CLPTM1L*	27	47	46	54	• (3)		• (6)	• (9)				5	
rs452932	1330253	*CLPTM1L*	28	47	47	54	• (6)	• (5)	• (8)					6	
rs452384	1330840	*CLPTM1L*	28	47	47	54	• (5)	• (3)	• (7)	• (16)	MYC			5	
rs467095	1336221	*CLPTM1L*	8	47	46	54				• (2)		POLR2A, ETS1		4	
rs31489	1342714	*CLPTM1L*	31	47	47	54					MEF2			—	•

^†^ r ^2^ ≥0.6, maximum inter-marker distance of 200kb and minimum MAF of 0.05;

AFR = African ancestry; EUR = European ancestry; AM = American ancestry; EA = East Asian ancestry;

Existence of a regulatory signature is indicated as dots (number of cell types this signature was observed, only indicated if occurring in ≥2 cell types);

RegulomeDB score indicates: 4 = TF binding + DNase peak, 5 = TF binding or DNase peak, 6 = motif hit, — = no data available;

Highlighted rows indicate that one or more surrogates for this SNP results in a likely functional consequence (RegulomeDB score of 2);

Mammal Conserv. = measurement of evolutionary placental mammal basewise conservation, the conserved sites are indicated.

### Potential regulatory changes

All previously reported cancer-associated SNPs and all possible surrogates at r ^2^ ≥0.6 were assessed for the presence of potential regulatory elements and evolutionary conservation among mammalian species (summarized in Table 4 and Supplementary Table 3). Surprisingly, none of the cancer-associated SNP surrogates were located in the coding regions of *TERT* or *CLPTM1L*. Many of these SNPs are associated with open chromatin (DNase hypersensitivity) and/or regulatory histone marks (H3K4Me1, H3K4Me3, H3K27Ac) in multiple cell types, alter known regulatory motifs and/or protein binding sites. One of the surrogate SNPs in the putative promoter region of *TERT*, rs2853669, is a conserved binding site for POLR2A, as were six other surrogate SNPs located intergenic between *TERT* and *CLPTM1L*, within the *CLPTM1L* gene region, and in the putative promoter region of *CLPTM1L*. One of the cancer-associated SNPs, rs2736098, and three surrogate SNPs in the 5’ region and putative promoter region of *TERT* were C>T SNPs located in the CpG island. Clusters of several surrogate SNPs located within *CLPTM1L* and just 3’ and 5’ of *CLPTM1L* were associated with many histone marks and open chromatin, and/or altered regulatory motifs and protein binding sites. None of the cancer-associated SNPs or their surrogates were associated with microRNA binding sites.

We used the RegulomeDB scoring system to compare and prioritize potential functional consequences of these SNPs. The cancer-associated SNPs in the 5’ region of *TERT*, most of the intergenic cancer-associated SNPs, and all the cancer-associated SNPs within *CLPTM1L* had surrogates with a likely functional consequence of affecting binding, indicated by a category 2 score (highlighted in Table 4 and Supplementary Table 3). None of the SNPs were identified to be associated with changes in expression of these genes.

## Discussion

Data from the 1000 Genomes Project ^34^ on 1627 variants at 5p15.33 for 1074 unrelated individuals were used to describe the population genetic patterns in this region. We evaluated differentiation among ancestral groups, allele frequency patterns, and the cancer-associated SNPs and surrogates for potential regulatory elements. We have previously shown that there is low nucleotide diversity and differentiation among populations in *TERT* and suggested that *TERT* may be constrained ^28, 29^; however, our previous population genetics study focused on telomere genes as a gene set and was limited to only four SNPs located within the *TERT* gene ^29^. In this study with better coverage of the *TERT-CLPTM1L* region, we determined that there is low nucleotide diversity across the 5p15.33 region in all ancestral groups and low differentiation among groups. As expected, African populations had more diversity, specifically at non-coding SNPs, compared to the other ancestral groups. However, East Asian populations had greater diversity at synonymous SNPs, and Europeans had the greatest frequency of non-synonymous changes. European and American ancestry individuals had very similar allele frequency patterns, as others have observed ^51^.

The significantly reduced normalized number of variant sites, heterozygosity, and MAFs, and low differentiation among ancestral groups for the coding sites, particularly for non-synonymous sites, compared with non-coding and silent changes suggests purifying selection in *TERT* and *CLPTM1*. African ancestry individuals had the greatest difference between the frequencies of non-coding *vs*. coding variants, consistent with stronger purifying selection; in contrast, European ancestry individuals had an excess of potentially deleterious non-synonymous SNPs. These observations are consistent with reports of genes important in cancer and complex disease ^42, 52– 54^ and recent genomic reports ^30– 33^. European ancestry individuals have been reported to have an excess of recently arisen potentially deleterious variants in disease genes ^33^. American and East Asian ancestry individuals also had an excess of coding variants compared to African ancestry individuals, suggesting weaker purifying selection in these populations as well. East Asian individuals had a particular excess of synonymous variants and very few non-synonymous variants. For the cancer-associated SNPs in this region, the risk allele was primarily the rare allele which additionally provides support for the hypothesis of constraint in this region. This evidence of purifying selection supports the importance of *TERT* and *CLPTM1* in disease, and the variation by ancestry suggests the level of selection differs by geographic region.

We found that several of the 23 SNPs that have been significantly associated with cancer at 5p15.33 [Reviewed in 25] had differing MAFs and heterozygosity among ancestral groups. Europeans and Americans had the most similar MAFs and heterozygosity estimates, which suggests significant admixture. These differences, reflected in the high *F*_ST_ values, may correlate to varying disease incidence rates among ancestral groups. For example, the breast cancer associated SNP, rs10069690 ^23^, had significantly different minor allele frequencies among ancestral groups; the homozygous risk allele genotype was significantly more common in African ancestry individuals (genotype frequency of 40% *vs*. 2.4% in East Asian, 6.8% in American, and 8.4% in European ancestry individuals) and less common in East Asian ancestry individuals. This difference may be associated with the higher incidence of breast cancer in African ancestry individuals (particularly for estrogen receptor-negative breast cancer) and lower incidence in East Asian individuals.

Many of the cancer-associated SNPs and surrogate SNPs were associated with potential regulatory elements, including histone marks, open chromatin, transcription factor binding sites, and/or regulatory motifs. There were only a few surrogates for the SNPs located within *TERT* and just 5’ of *TERT* due to the low levels of LD in these regions; and, there were a large number of surrogates for the SNPs located close to and within *CLPTM1L* where LD was strong and recombination low, most of these surrogates were shared among the cancer-associated SNPs in this region. Many of the surrogate markers were located in the putative promoter regions of *TERT* and *CLPTM1L* and may affect gene regulation. The RegulomeDB scoring approach allowed us to classify variants based on all of the regulatory information. This approach determined that surrogate SNPs for many of the cancer-associated SNPs are functional variants with a likely role in regulation; these should be prioritized for functional assays.

## Conclusions

Our analysis of diversity in this important cancer-associated region of 5p15.33 provides background information for understanding variation in the general population. The functional impact of common variation in this region needs to be examined experimentally, but we could speculate that the diversity of coding variants among different ethnicities could have mild effects on the phenotype disparity observed among these populations. Many of the cancer-associated SNPs and/or surrogates at 5p15.33 are associated with regulatory changes and candidates for evolutionary selection. Evidence of purifying selection in *TERT* and *CLPTM1L* highlights their functional importance and associations with complex disease. We have identified SNPs in this region that are likely involved in regulation of the *TERT* and/or *CLPTM1* genes. Future studies of the functional consequences of the 5p15.33 variants will be required to understand their contribution to cancer etiology.

## Data availability

The data referenced by this article are under copyright with the following copyright statement: Copyright: © 2014 Mirabello L et al.

Data associated with the article are available under the terms of the Creative Commons Zero "No rights reserved" data waiver (CC0 1.0 Public domain dedication). 

F1000Research: Dataset 1. Genotype data for 1627 variants on 5p15.33 (hg19, chr5: 1,243,287–1,355,002) for 1074 individuals from 14 populations, 10.5256/f1000research.5186.d35521 ^55^

## Supplementary material

**Supplementary Table 1.** Recombination hotspot inference summary.

.

**Supplementary Table 2.** All possible surrogate markers by ancestral group and their rank for the 23 cancer-associated SNPs based on a R ^2^ ≥0.60, maximum inter-marker distance of 200kb, and minimum MAF of 0.05.

.

**Supplementary Table 3.** Previously reported multiple-cancer susceptibility loci at 5q15.33 and their surrogates at an r ^2^ ≥0.6 and regulatory elements.

.

**Supplementary Table 4.** Risk allele frequencies of the cancer-associated SNPs at the *TERT-CLPTM1L* locus by population.

.

**Supplementary Figure 1.** Summary of population genetics parameters in East Asian ( **A.**) and American ( **B.**) ancestry individuals for 5p15.33.

.

Linkage disequilibrium (LD), recombination hotspots, heterozygosity, and pairwise *F*st values are shown for the cancer-associated SNPs (red dots), surrogate SNPs (blue dots), and non-surrogate SNPs (grey dots). LD pattern (see color legend) is shown for SNPs with a MAF ≥0.05. The red lines represent an extension of the location of the cancer-associated SNPs. The blue lines in the heterozygosity plot indicate the location of the recombination hotspots. For the pairwise *F*st estimates, the populations are indicated in the top corner of each graph. AFR = African ancestry; EUR = European ancestry; AM = American ancestry; ASN = East Asian ancestry.
