# Supplementary figures and images for: Characterization of population-based variation and putative functional elements for the multiple-cancer susceptibility loci at 5p15.33
Source: F1000Res. 2014 Oct 2;3:231. [Version 1] doi: 10.12688/f1000research.5186.1 (PMC4654438; doi:10.12688/f1000research.5186.1)

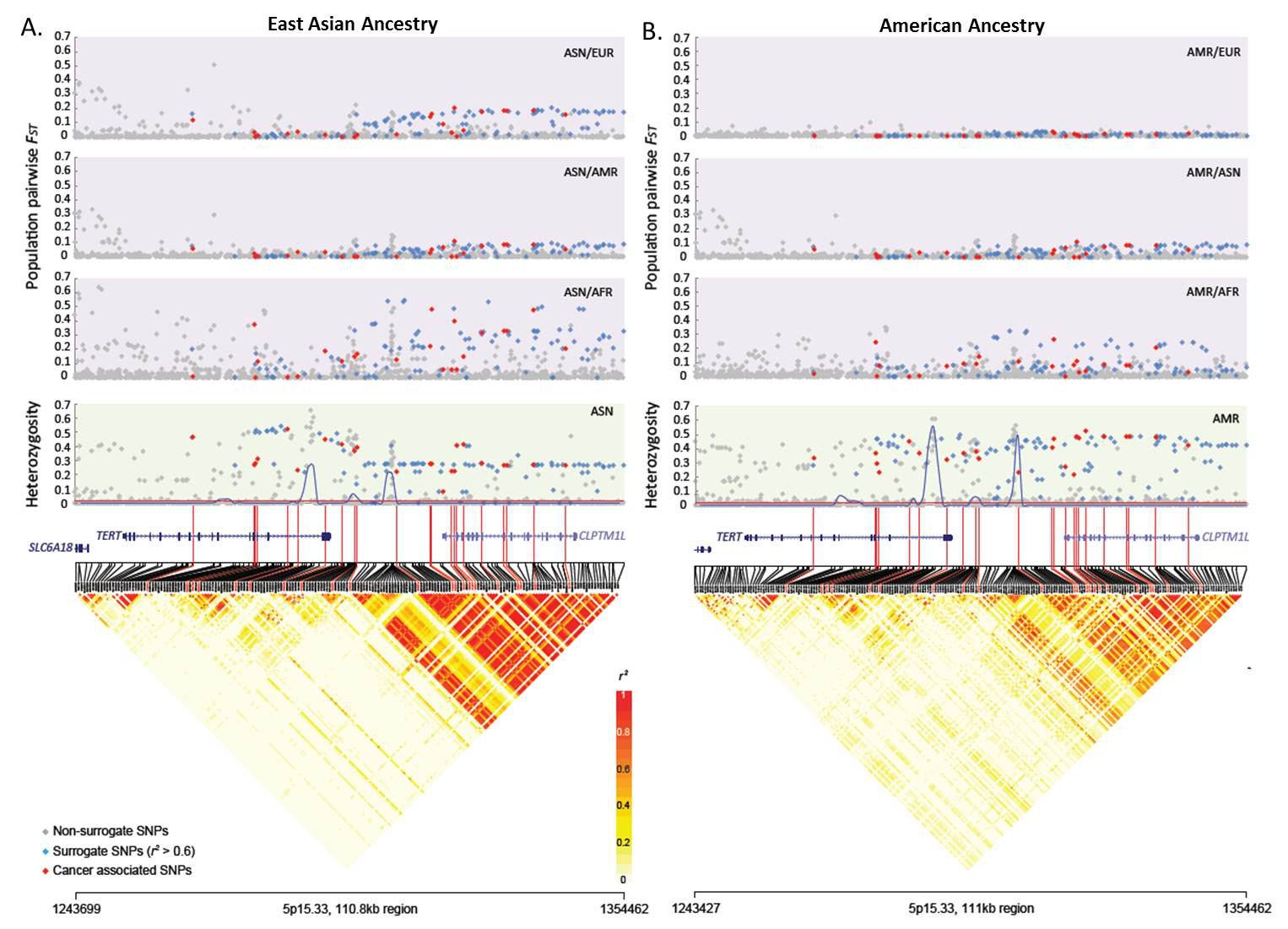

Supplement: Supplementary file 6 [file f1000research-3-5532-s0005.tgz › c54e6a31-d05e-496e-b35c-ea848b6bc10f.tif]
