# Supplementary material for: Characterization of population-based variation and putative functional elements for the multiple-cancer susceptibility loci at 5p15.33
Source: F1000Res. 2014 Oct 2;3:231. [Version 1] doi: 10.12688/f1000research.5186.1 (PMC4654438; doi:10.12688/f1000research.5186.1)
Supplement: Peer review history

# Peer review: Referee response for version 1

**DOI:** 10.5256/f1000research.5532.r9055

**Published:** 2015-06-16

**Authors:** John L. Hopper, Miroslav K. Kapuscinski

**Affiliations:** 1 Centre for Molecular, Environmental, Genetic and Analytic Epidemiology, University of Melbourne, Carlton, Vic, Australia; 2 Centre for Epidemiology and Biostatistics Melbourne School of Population and Global Health, University of Melbourne, Carlton, Vic, Australia

Mirabello *et al *present here a comprehensive bioinformatics investigation of genetic variation at the telomerase-containing locus (5p15.33) that has been associated with a range of malignancies. Given high biological plausibility of telomerase involvement in cancer pathology, this is an important study that could assist in further research on this putative susceptibility locus.

The research strategy described in this well written paper should be applauded as it can be easily applied to other genomic regions of interest and provides an excellent example of extracting more useful information from existing data. In particular, the use of 1000 Genomes data provides an opportunity to examine the distribution of a wider range of variants in detail not possible using GWAS genotyping alone.

As the authors point out, highly significant associations of a number of SNP variants are paralleled by rather small phenotypic associations with these variants. The most common protein altering variant (rs61748181) identified in the available data appears to have modest associations. This is not a unique situation and it makes choosing variants for functional characterization difficult considering the investment required for such comprehensive studies. It should be stressed that direct identification of causal variants from GWAS data has not been very successful. The present report demonstrates the need for well-designed analytical approach based on the sequence information (1000 Genomes) together with other data (ENCODE) to reveal credible causal candidates and narrow the choices for subsequent experimental verification. The authors acknowledge the key role of future functional work in this discovery process.

As the data from 1000 Genomes Consortium comes from unaffected people inclusion of other information in the analytical pipeline that allows comparison of germline and tumour sequence information (e.g. The Cancer Genome Atlas, eQTLs) might allow further refinement of variant evaluation with different mechanisms evident in different cancers (e.g. relevance of promoter mutations - Lindner *et al., *2015 and Spiegl-Kreinecker *et al., *2015).

The evidence for purifying selection in TERT-CLPTM1L region points to the importance of maintaining the structural integrity of this locus but also suggests that mechanisms other than protein altering mutations may play significant role such as interactions with other genes such as MYC ( Koh *et al.*, 2015) or miR-34a ( Xu *et al., *2015).

The rationale for setting the threshold for marker surrogacy at r ^2^ = 0.6 (p7) while using r ^2^ = 0.8 for LD calculations (p3) should be explained.

In summary, this is well designed and presented study that demonstrates the potential of using high throughput sequencing data together with growing resources such as ENCODE to enhance understanding of traditional genome-wide genotyping experiments. The title reflects well the contents, the abstract is appropriate and occlusions are justified and balanced.

We have read this submission. We believe that we have an appropriate level of expertise to confirm that it is of an acceptable scientific standard.

# Peer review: Referee response for version 1

**DOI:** 10.5256/f1000research.5532.r6299

**Published:** 2014-10-10

**Authors:** Duncan Baird

**Affiliations:** 1 Institute of Cancer and Genetics, Cardiff University, Cardiff, UK

Numerous studies have identified variation at the TERT-CLPTM1L locus in conferring an increased risk of many different cancer types.

Here the authors have examined the genetic architecture of the TERT-CLPTM1L locus using sequence data from the 1000 genomes project.  Given the potential significance of this locus, this type of work is important as it has the potential to identify functional variants that might not have been uncovered with the various GWAS undertaken to identify risk variants.  Thus far none of the risk variants identified at this locus with GWAS results in non-synonymous protein changes, however this study provides data to indicate that some of these variants may be associated with regulatory sequences and chromatin marks.  This study also identified 26 variants that result in non-synonymous protein changes in the hTERT or the CLPTM1L genes.

This is a well written manuscript and the conclusions are appropriately backed up by the data provided.  The title is appropriate and the abstract adequately summarises the article.  Overall this manuscript provides useful information that that will underpin future work to establish the importance of this locus in conferring cancer risk.

I have no major criticisms of this work; however I recommend that a more rigorous statistical review, than I am able to provide, is undertaken of this manuscript.

I have read this submission. I believe that I have an appropriate level of expertise to confirm that it is of an acceptable scientific standard.
